# Large‐Area Deposition of Highly Crystalline F4‐Tetracyanoquinodimethane Thin Films by Molecular Step Templates

**DOI:** 10.1002/smsc.202400038

**Published:** 2024-04-12

**Authors:** Fengquan Qiu, Wei Deng, Xinmin Shi, Dewen Ai, Xiaobin Ren, Anyi Dong, Xiujuan Zhang, Jiansheng Jie

**Affiliations:** ^1^ Institute of Functional Nano & Soft Materials (FUNSOM) Jiangsu Key Laboratory for Carbon‐Based Functional Materials & Devices Soochow University Suzhou Jiangsu 215123 P. R. China; ^2^ Macao Institute of Materials Science and Engineering (MIMSE) MUST‐SUDA Joint Research Center for Advanced Functional Materials Macau University of Science and Technology Taipa Macau SAR 999078 P. R. China

**Keywords:** crystalline organic thin films, fluorinated tetracyanoquinodimethane, n‐channel organic semiconductors, organic complementary inverters, organic thin‐film transistors

## Abstract

Theoretical studies have unequivocally determined the exceptional electron transport properties of the fluorinated tetracyanoquinodimethane (F*x*‐TCNQ) family, presenting a promising avenue for the realization of high‐performance n‐channel organic thin‐film transistors (OTFTs). However, owing to the intrinsic low crystallinity of this class of materials, F*x*‐TCNQ‐based n‐channel OTFTs have not been experimentally achieved so far. Herein, a molecular step template (MST)‐assisted method that dramatically improves the crystallinity of F4‐TCNQ thin films is reported. The MST not only lowers the nucleation barrier of F4‐TCNQ molecules along the in‐plane direction but also reduces the nucleation density. This approach facilitates the realization of compact, oriented, and highly crystalline F4‐TCNQ thin films, resulting in impressive electron mobility of up to 2.58 cm^2^ V^−1^ s^−1^. Notably, this achievement surpasses the electron mobility of F4‐TCNQ thin films fabricated without the MST by a factor of 10^7^. Furthermore, the incorporation of the p‐type MST provides a novel pathway for constructing complementary inverters, showcasing a high voltage gain of 112.6 V V^−1^ and a substantial noise margin of 89.3% with exceptional uniformity. In this work, a general and efficient route is paved to produce high‐performance n‐channel OTFTs toward organic complementary circuits.

## Graphical abstract

A molecular step template‐assisted method is proposed to overcome intrinsic limitations of low‐crystallinity nature of high‐mobility n‐type fluorinated tetracyanoquinodimethane (F*x*‐TCNQ) molecules. This approach enables to yield of large‐area, fully connected, and highly crystalline F4‐TCNQ thin films, showing exceptionally high electron mobility of 2.58 cm^2^ V^−1^ s^−1^ and achieving high‐performance inverters.

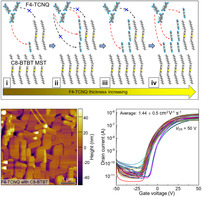

## 1 Introduction

Highly crystalline thin films of organic semiconductors (OSCs) have emerged as pivotal elements in the field of organic electronics and optoelectronics and have drawn considerable attention over the years.^[^ ^1^, ^2^, ^3^, ^4^, ^5^ ^]^ In contrast to their amorphous counterparts, these crystalline thin films possess outstanding comprehensive properties, such as larger grain dimension, lower defect density, well‐ordered molecular arrangements, etc., which are key requirements for fundamental material studies.^[^ ^6^ ^]^ Moreover, highly crystalline OSC thin films significantly enhance device performance and stability, particularly in terms of carrier mobility in organic thin‐film transistors (OTFTs). To date, diverse fabrication techniques have been developed, yielding large crystalline thin films of OSCs with coverage area up to 100 cm^2^ and high hole mobility over 10 cm^2^ V^−1^ s^−1^ that exceeds the performance of commercial amorphous silicon thin films.^[^ ^2^, ^7^, ^8^, ^9^, ^10^, ^11^, ^12^, ^13^, ^14^, ^15^ ^]^ The expansive area and superior electrical performance of these crystalline OSC thin films pave the way for the ideal production of thin‐film electronics. Unfortunately, the majority of the reported crystalline OTFTs (C‐OTFTs) are predominantly p channels, and the progress in developing n‐channel C‐OTFTs lags significantly behind that of their p‐channel counterparts.^[^ ^16^, ^17^, ^18^ ^]^ Given the practical demands for complementary circuits, the quest for novel n‐type crystalline OSC thin films remains of paramount significance.

The fluorinated tetracyanoquinodimethane (F*x*‐TCNQ) family stands as a venerable and classic organic semiconducting materials that propel advancements in (opto)electronics and photonics.^[^ ^19^, ^20^, ^21^, ^22^ ^]^ Their allure stems from a high electron affinity, designating them as p‐type molecular dopants for modulating the electrical properties of OSCs,^[^ ^23^, ^24^ ^]^ inorganic compound semiconductors,^[^ ^25^, ^26^ ^]^ 2D semiconductors,^[^ ^19^, ^27^ ^]^ and beyond. Additionally, F*x*‐TCNQ, enriched with fluorine atoms, can lower the lowest unoccupied molecular orbital (LUMO) energy level and enhance ambient stability, rendering it a promising candidate for high‐performance n‐channel OTFTs. Despite the theoretical confirmation of the high electron transport properties of F*x*‐TCNQ,^[^ ^28^, ^29^ ^]^ the experimental realization of its OTFTs remains an uncharted territory, primarily attributed to the challenges in achieving large‐grain, fully connected, and highly crystalline F4‐TCNQ thin films. The low degree of crystallinity of F4‐TCNQ molecules presents formidable hurdles in translating their theoretical promise into experimental fruition.

In this work, we report an efficient molecular step template (MST)‐assisted method to fabricate large‐area and highly crystalline F4‐TCNQ thin films with fully connected grains. A single‐crystalline 2,7‐dioctyl[1]‐benzothieno[3,2‐b][1]benzothiophene (C8‐BTBT) film with a unique molecular step structure served as an underlayer template is introduced to lower the nucleation barrier along the in‐plane direction and nucleation density of F4‐TCNQ molecules. Therefore, complete and crystalline F4‐TCNQ thin films with micrometer‐scale and compact grains are formed by sequential evaporation of F4‐TCNQ. OTFT arrays with the crystalline F4‐TCNQ thin films exhibit a maximum electron mobility of 2.58 cm^2^ V^−1^ s^−1^ in the ambient condition, which is higher by a factor of 10^7^ than that of thin films without the use of the MST, simultaneously show a good air stability over 1 month. Furthermore, by combining the p‐channel OTFT made from the underlayer C8‐BTBT MST and the n‐channel OTFT made from the upper F4‐TCNQ, organic complementary circuits with average gain up to 76 ± 14.7 V V^−1^ as well as noise margin of 72.4% ± 7.5% are readily attainable. This MST‐assisted crystallization methodology overcomes the bottleneck of low crystallinity of high‐mobility n‐type OSCs and may lead to practical technologies compatible with organic circuits.

## 2 Results and Discussion

### 2.1 Morphologies and Structures of F4‐TCNQ Thin Films Grown on the MST

**Figure 1:**
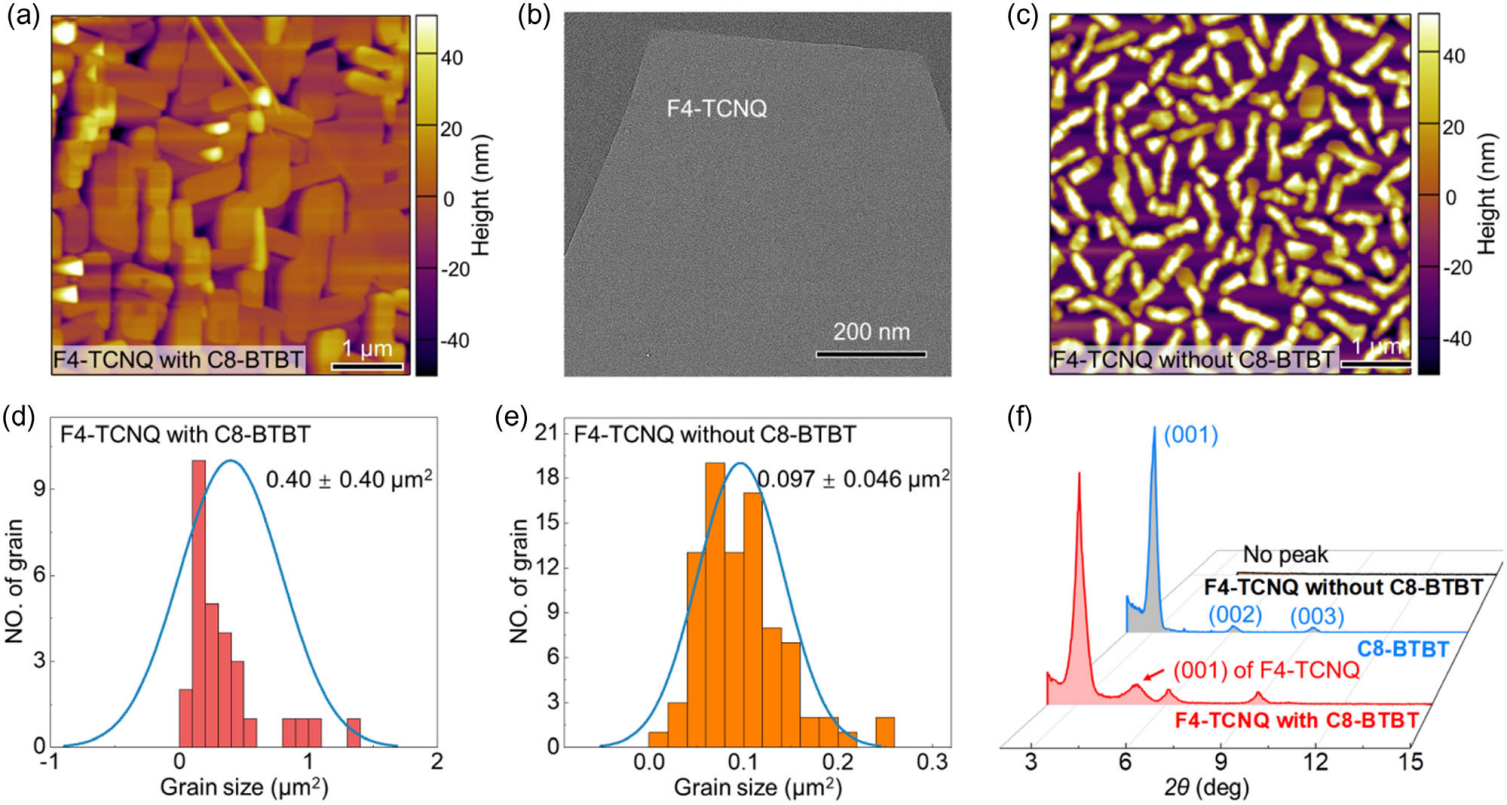
a) Schematic diagram of the proposed MST‐assisted method for fabricating highly crystalline F4‐TCNQ thin films. b,c) AFM topography of the resulting F4‐TCNQ thin films with and without MST. The inset in (b) is a TEM image of the crystal domain in the F4‐TCNQ thin films with MST. d,e) Histogram of the average size of grains within the F4‐TCNQ thin films with and without MST. f) XRD patterns of pure C8‐BTBT MST and F4‐TCNQ thin films with and without MST.

As a molecular template, its surface smoothness and quality are crucial for subsequent F4‐TCNQ growth. Hence, we selected grain‐boundary‐free and smooth C8‐BTBT single‐crystalline films (C8‐BTBT SCF) with step‐and‐terrace structures as underlayer substrates (Figure S1, Supporting Information). The C8‐BTBT SCF was fabricated according to our previous report.^[^ ^30^ ^]^ After thermal evaporation of 18 nm of F4‐TCNQ on the C8‐BTBT SCF, compact and large crystalline grains with sheetlike shapes were clearly observed on the C8‐BTBT SCF, and the resulting F4‐TCNQ thin film presented high crystallinity (**Figure** **1**a). Transmission electron microscopy (TEM) further confirmed that there were no detectable grain boundaries or defects in the F4‐TCNQ crystal domain (Figure 1b). This morphology is very different from that of F4‐TCNQ thin films directly grown on a bare divinyltetramethyldisiloxane bis(benzocyclobutene) (BCB) substrate without C8‐BTBT SCF; these thin films have small grains and rough surfaces with a root mean square of 26.77 nm (Figure 1c). We performed a statistical analysis of the size of the grains within the F4‐TCNQ thin films in an area of 5 × 5 μm. The average grain size of the F4‐TCNQ thin films grown on the MST reaches 0.40 ± 0.40 μm^2^ (Figure 1d), which is at least four times greater than that of the F4‐TCNQ thin films without the use of the MST (Figure 1e). The significantly increased grain size is indicative of an enhancement in the 2D in‐plane growth mode of F4‐TCNQ molecules in the presence of the C8‐BTBT SCF underlayer, while effectively decreasing the density of grain boundaries and enhancing the continuity between crystal domains to achieve high device performance.

To obtain structural information for the crystalline F4‐TCNQ thin films, out‐of‐plane X‐ray diffraction (XRD) measurements were carried out. The XRD pattern of the F4‐TCNQ thin films on the MST shows sharp and strong peaks (Figure 1f). The diffraction peaks at 2*θ* = 3.06°, 6.04°, and 9.06° correspond to the (00l) reflection of the C8‐BTBT SCF; thus, we can deduce that the C8‐BTBT molecules have a standup orientation according to the C8‐BTBT single‐crystal data, which is consistent with previous reports.^[^ ^6^, ^31^ ^]^ The diffraction peak at 2*θ* = 5.02° corresponds to the (001) plane of F4‐TCNQ.^[^ ^32^ ^]^ The appearance of only one intense out‐of‐plane reflection suggested that the F4‐TCNQ crystalline grains exhibited an ordered stack structure in the vertical direction and the F4‐TCNQ molecules tend to stand on the C8‐BTBT MST. The aforementioned morphological and structural results demonstrated that the molecular template not only improved the morphology but also increased the orientation of the F4‐TCNQ thin films.

### 2.2 Formation Mechanism of F4‐TCNQ Thin Films on C8‐BTBT Surfaces

**Figure 2:**
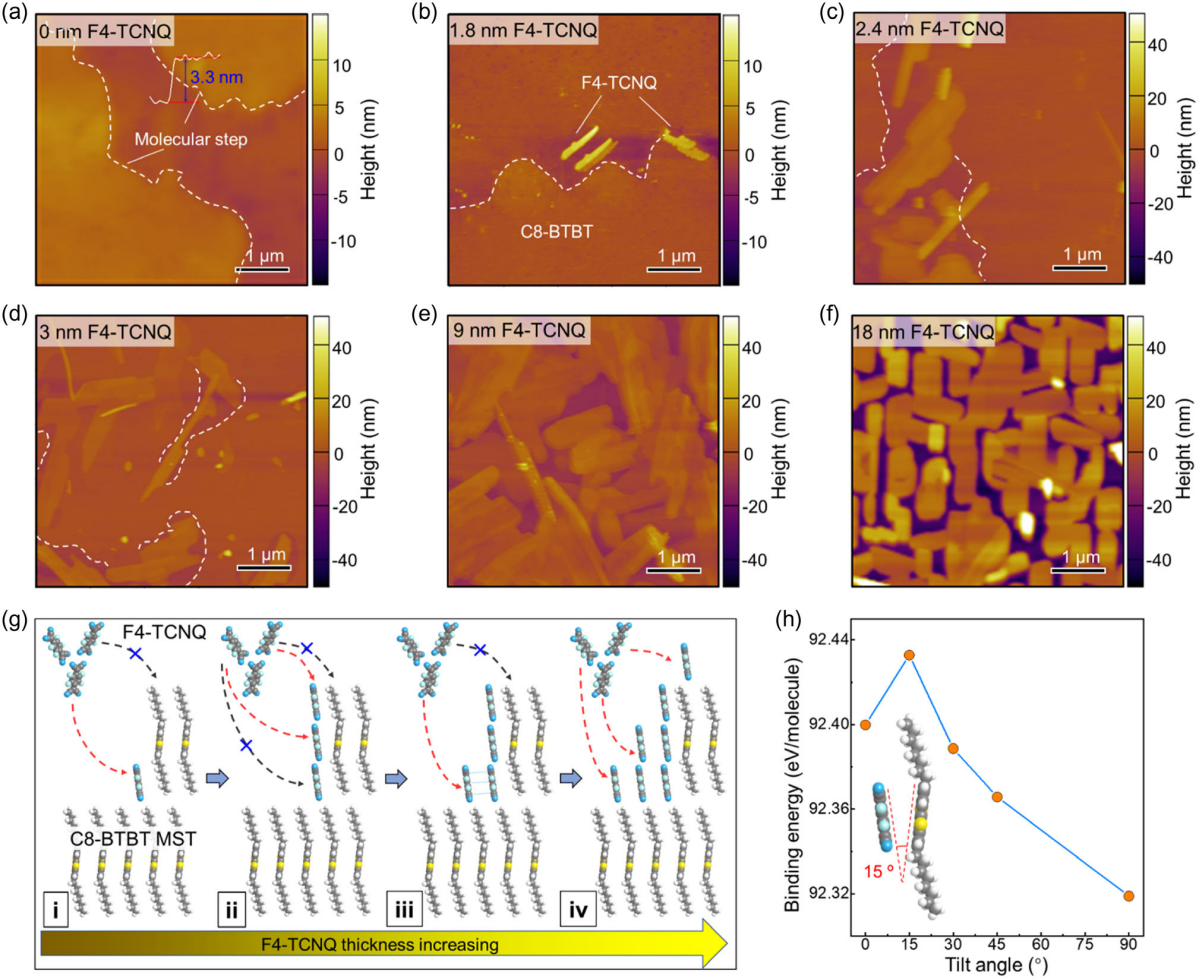
a–f) Sequence of AFM images showing the F4‐TCNQ growth trend on the MST. g) Schematic representation of the nucleation and growth of F4‐TCNQ molecules on the C8‐BTBT SCF. h) DFT calculations of the structures of the F4‐TCNQ molecule near the molecular step and binding energy per molecule for the different configurations.

To elucidate the formation mechanism, we first studied the morphological evolution of F4‐TCNQ thin films on C8‐BTBT SCFs by atomic force microscopy (AFM) imaging. We found that the C8‐BTBT SCF template exhibited crystallographic step edges with a height of approximately 3.3 nm (**Figure** **2**a and S2a, Supporting Information), which is consistent with the thickness of one molecular layer of C8‐BTBT. At the early stage of thin‐film growth (1.8 nm adlayer), the evaporated F4‐TCNQ molecules preferentially adsorb onto the step edges (Figure 2b and S2b, Supporting Information), which can serve as nucleation sites. With the addition of a 2.4 nm thick layer, F4‐TCNQ grew around the preformed crystal nucleus near the step edges rather than on the template surface (Figure 2c). This difference is attributed to the atomically smooth C8‐BTBT SCF layer, which cannot offer additional nucleating points. In this scenario, the added molecules preferentially deposit along the lateral direction, which is strong evidence for the reduced nucleation barrier of 2D F4‐TCNQ growth. Because surface energy of the F4‐TCNQ and C8‐BTBT is largely the same (Figure S3 and Table S1, Supporting Information), surface energy is unable to explain the change of F4‐TCNQ molecular arrangement on the C8‐BTBT SCF. With the gradual addition of additional molecules, the crystalline grains grow continuously and coalesce to form compact connections, as shown in Figure 2d–f.

On the basis of the morphology evolution and XRD results, we propose the mechanism in Figure 2g for the growth of F4‐TCNQ on C8‐BTBT via MST. First, due to the high surface energy at the molecular step edges,^[^ ^33^ ^]^ F4‐TCNQ molecules selectively aggregate and stand out here through *π*–*π* interactions between C8‐BTBT and F4‐TCNQ molecules (Figure 2g–i). To determine this molecular arrangement, we performed density‐functional theory (DFT) studies. According to the possible adsorption sites of F4‐TCNQ molecules, we constructed a series of possible molecular configurations at the molecular step edges, where the F4‐TCNQ molecule unit was leaning toward C8‐BTBT by different tilt angles (Figure S4, Supporting Information). We found that when the tilt angle between the cyclohexadiene of F4‐TCNQ and the benzothiophene ring of C8‐BTBT is ≈15°, the configuration is the most thermodynamically stable (Figure 2h). The DFT calculations further validated our hypothesis at the initial stages of F4‐TCNQ growth. In the next step, because the length of the F4‐TCNQ molecule is less than the height of the C8‐BTBT molecular step, the step still can play a significant role. The adsorbent molecules would continually stack above the deposited F4‐TCNQ molecule rather than on the alkyl chains of C8‐BTBT. Subsequently, the adsorbent molecules preferentially attach to the side of the pre‐deposited F4‐TCNQ molecules rather than to the C8‐BTBT surface (Figure 2g–iii) because the C–H…N hydrogen bond interactions between F4‐TCNQ and C8‐BTBT are weaker than the *π*–*π* interactions between adjacent F4‐TCNQ molecules, C8‐BTBT and the added F4‐TCNQ. With the deposition of F4‐TCNQ, the *π*–*π* interactions between the exposed conjugated rings of C8‐BTBT and the F4‐TCNQ molecule are shielded; thus, the added F4‐TCNQ molecules tend to stack upward (Figure 2g–iv). Finally, with a further increase in the amount of added F4‐TCNQ molecules, the crystalline grains merge with the ones to form connective films.

The proposed MST‐assisted method should be broadly applicable to other OSCs because the molecular template could offer molecular steps as preferential nucleation sites to facilitate crystallization. To demonstrate the universal compatibility of our methodology, another F*x*‐TCNQ family member, such as F6‐TCNNQ, was tested. Similarly, the C8‐BTBT MST enabled the formation of highly crystalline F6‐TCNNQ thin films with large grains, whereas the thin films deposited without MST resulted in small and unconnected grains under the same evaporation deposition process (Figure S5, Supporting Information).

### 2.3 Performance and Stability of the F4‐TCNQ OTFTs

**Figure 3:**
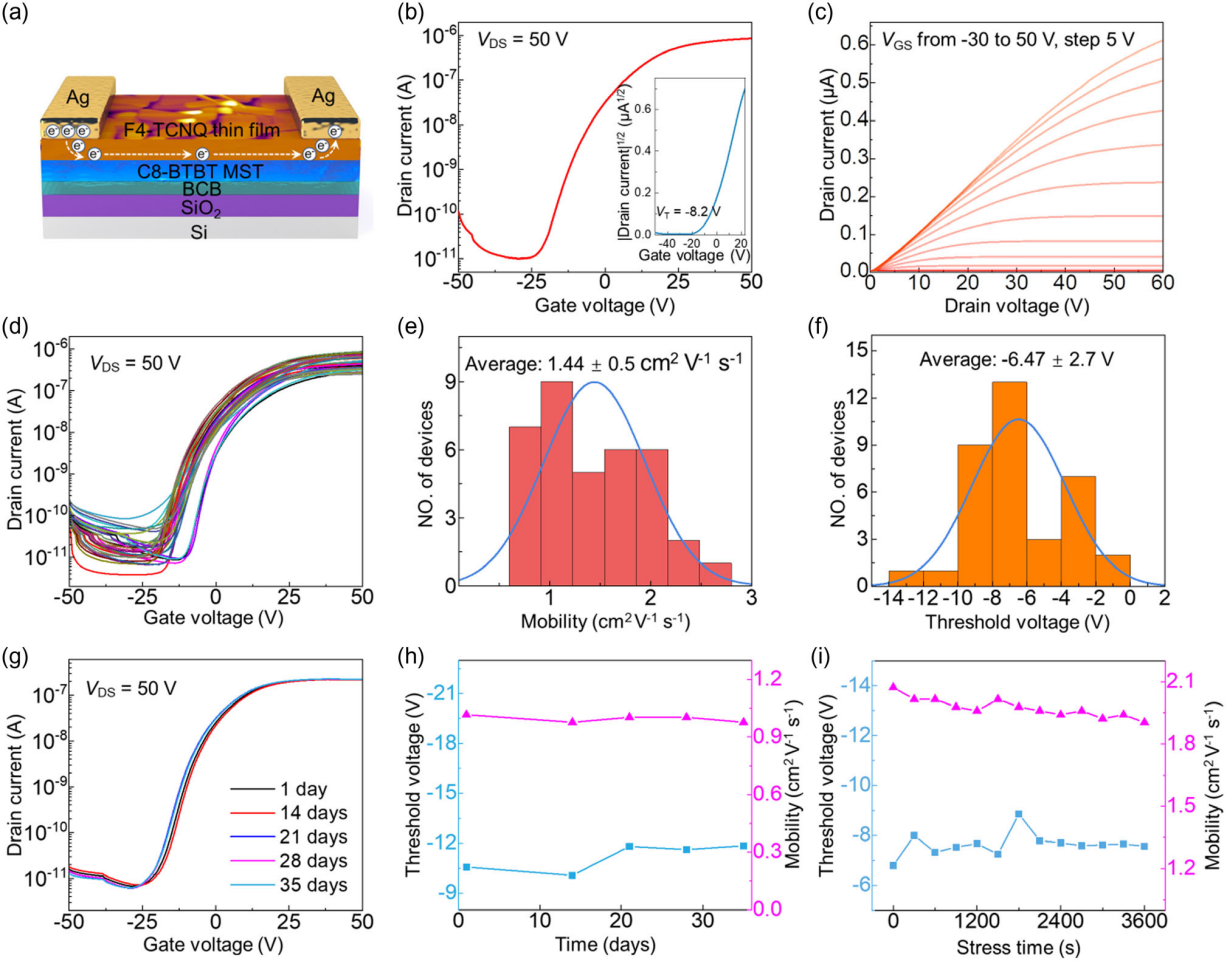
a) Device schematic of an F4‐TCNQ thin film on a C8‐BTBT MST. Typical transfer b) and output c) characteristics of the F4‐TCNQ crystalline thin‐film‐based OTFT. The inset in (b) shows the *V*_th_ of the device. d) Transfer characteristics of 6 × 6 OTFT arrays. e,f) Statistical distribution of mobility and *V*_th_. g) Transfer characteristics of an OTFT fabricated with F4‐TCNQ on MST as‐prepared and after different storage times. h) *V*_th_ and mobility shift versus storage time. i) *V*_th_ and mobility shift versus stress time.

The successful formation of continuous and highly crystalline F4‐TCNQ thin films enables their application in high‐performance n‐channel OTFTs. Top‐contact bottom‐gate OTFTs with an F4‐TCNQ thin film on C8‐BTBT SCF as the active layer were fabricated. In this structure, C8‐BTBT MST served as the dielectric layer and electrons transported at the interface of C8‐BTBT and F4‐TCNQ thin film, as shown in **Figure** **3**a. More importantly, C8‐BTBT without active functional groups as electron trap sites can enhance the electron mobility. For comparison, an F4‐TCNQ OTFT without the C8‐BTBT molecular template was also fabricated as a reference device. Figure 3b shows representative drain current versus gate voltage (*I*_DS_ *–V*_GS_) characteristics for OTFTs made from F4‐TCNQ with the C8‐BTBT molecular template. With the assistance of the C8‐BTBT molecular template, the F4‐TCNQ thin film exhibits n‐type field‐effect conduction with a high on/off current (*I*_ON_ */I*_OFF_) ratio >10^4^, whereas the F4‐TCNQ OTFT without C8‐BTBT (Figure S6, Supporting Information) displays negligible n‐type field‐effect conduction properties and a very small *I*_ON_ */I*_OFF_ ratio of ≈2.0. The ON‐state current at the same *V*_GS_ and *V*_DS_ values is more than four orders of magnitude greater than that for the reference device. Compared with that of the reference device, the electron mobility of F4‐TCNQ OTFTs with the C8‐BTBT molecular template improved markedly, reaching a high mobility of 2.58 cm^2^ V^−1^ s^−1^, which is at least 10^7^ times greater than that of the reference device (2.12 × 10^−7^ cm^2^ V^−1^ s^−1^). The drain current–drain voltage (*I*_DS_ *–V*_DS_) characteristics in Figure 3c also reveal that the use of an MST substantially increases the output current. Apart from the improvement in the mobility, we note that the F4‐TCNQ OTFT shows a negative threshold voltage (*V*_th_) and is a normally on device, which is expected to be the result of charge transfer between C8‐BTBT and F4‐TCNQ. Since the ionization potential of C8‐BTBT appears to be almost equivalent to the electron affinity of F4‐TCNQ,^[^ ^24^, ^34^ ^]^ spontaneous charge transfer would take place only to fill the trap states near the LUMO level of F4‐TCNQ, producing a trap healing effect and leading to a negative *V*_th_ shift. The efficient charge transfer was confirmed by ultraviolet–visible (UV–Vis) absorption spectroscopy (Figure S7, Supporting Information), which revealed an additional absorption peak corresponding to singly charged F4‐TCNQ anions.

Furthermore, we fabricated 6 × 6 OTFT arrays to verify the uniformity of the resulting F4‐TCNQ thin films made from C8‐BTBT MST. All the devices worked well (100% yield) and exhibited almost identical transfer characteristics (Figure 3d). The statistical results for the 36 OTFTs on the same substrate were further evaluated (Figure 3e,f). The electron mobility was on average 1.44 ± 0.5 cm^2^ V^−1^ s^−1^, which is markedly higher than that achieved for amorphous silicon transistors (0.5–1 cm^2^ V^−1^ s^−1^).^[^ ^35^, ^36^ ^]^ Importantly, to the best of our knowledge, this is the first report of an F4‐TCNQ OTFT. The average *V*_th_ exhibited a distribution of −6.47 ± 2.7 V, a deviation of only 5.4% of the working gate voltage window (50 V). Figure S8, Supporting Information, plots the electrical characteristics in two dimensions with the intensity of the color, indicating a random variation in performance. We noted that the mobility standard deviation is relatively high; this is likely to be mainly due to the presence of grain boundaries. In addition, deviations in device dimensions such as the channel length and width are also responsible for some of the variation in performance.

It is well known that stability is a basic requirement for realistic application. On the molecular level, F4‐TCNQ has a low LUMO and multiple fluoroalkyl chains; thus, we believe that F4‐TCNQ should exhibit good stability. Figure 3g shows the shelf stability of representative F4‐TCNQ OTFTs that were tested over a period of 1 month under ambient conditions (humidity ≈10% and temperature ≈290 K). No appreciable changes in the transfer curves were observed. The *V*_th_ shift was 1.28 V, and the electron mobility changed by only ≈4%, as depicted in Figure 3h, demonstrating remarkable air stability. When the device was stored in a moist environment (humidity 50%–70%), it was found that the performance of the F4‐TCNQ OTFT changed only slightly (Figure S9, Supporting Information). Furthermore, the bias stability of the F4‐TCNQ OTFT was checked by repeated measurements. Electrical stress was applied under *V*_GS_ = *V*_DS_ = 50 V in air, and the transfer characteristics of the device before and after stress were measured. The *I*_DS_ *–V*_GS_ curves showed no appreciable changes over a time scale of up to 3600 s (Figure S10, Supporting Information). Both *V*_th_ and mobility exhibited very small magnitudes of change (Figure 3i). In addition, the F4‐TCNQ OTFT with the C8‐BTBT molecular template also displayed remarkable operating cycle stability when switched continuously ON‐ and OFF‐state conditions over a period of 14 000 s (Figure S11, Supporting Information). These data illustrate that the F4‐TCNQ material indeed has superior stability, as expected.

### 2.4 Performance of Complementary Inverters

**Figure 4:**
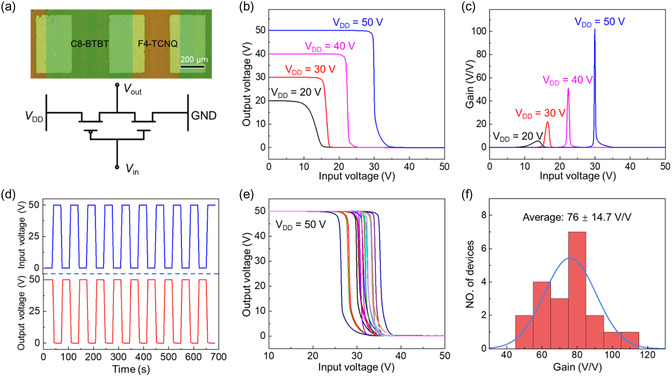
a) Optical microscope image and circuit diagram of the complementary inverters based on the C8‐BTBT MST and F4‐TCNQ thin films. b) VTC of the inverter at different supply voltages (*V*_DD_). c) Gain as a function of the input voltage for different supply voltages. d) Output characteristics of the inverter as a NOT gate. e) VTC of the 20 inverters on the same substrate. f) Statistical histogram of the gain values measured from the 20 inverters.

Due to their high mobility and excellent air stability, our F4‐TCNQ OTFTs have great potential for practical application. Given the p‐channel property of the underlayer C8‐BTBT SCF, we combined the F4‐TCNQ OTFT with the C8‐BTBT OTFT to demonstrate the complementary inverters. To fabricate the inverter, we used a shadow mask to selectively deposit F4‐TCNQ molecules on the C8‐BTBT SCF, forming alternating patterns of F4‐TCNQ thin films and C8‐BTBT SCFs. Optical microscopy images and circuit schematics of the inverter are shown in **Figure** **4**a, in which the green and yellow–brown regions are the C8‐BTBT p‐channel and F4‐TCNQ n‐channel, respectively. The voltage‐transfer characteristics (VTCs) of a typical inverter exhibit sharp switching with rail‐to‐rail output swings, as displayed in Figure 4b. The values of the calculated voltage gain and static noise margin (SNM) are 102.1 V V^−1^ and 78.9%, respectively, at a supply voltage (*V*_DD_) of 50 V. The details on the extraction of the SNM from the VTC can be found in Figure S12, Supporting Information.^[^ ^37^ ^]^ Figure 4c shows that the measured gain increases with increasing *V*_DD_; we could still achieve a satisfactory gain at low *V*_DD_. The complementary inverter also has the correct NOT logic function. After several tens of cycles and 700 s of continuous operation, no degradation of the NOT function was noted in ambient air (Figure 4d), indicating sufficient durability of the circuit.

Finally, we constructed 20 inverters on a centimeter scale to verify the uniformity of the performance (Figure S13, Supporting Information). All the inverters exhibit standard VTC, and the voltage gain values are all in excess of 52.7 V V^−1^ (Figure 4e). The average voltage gain is 76 ± 14.7 V V^−1^, and the best value reaches 112.6 V V^−1^ (Figure 4f), which is much greater than that of most of the reported organic inverters.^[^ ^38^, ^39^, ^40^, ^41^, ^42^, ^43^, ^44^, ^45^, ^46^ ^]^ Notably, the voltage gain can be further improved by optimizing the dimensions of C8‐BTBT OTFTs because C8‐BTBT OTFTs have greater mobility than F4‐TCNQ OTFTs (Figure S14, Supporting Information) and can lead to an unbalanced current. In addition, the maximum and average SNM values for the 20 inverters (Figure S15, Supporting Information) can reach 89.3% and 72.4% ± 7.5%, respectively, which are greater than those of most organic inverters. The good and uniform performance of the complementary inverters proves that our method is ready for the achievement of more complicated logic circuits, such as ring oscillation.

## 3 Conclusion

In summary, we demonstrated a novel and universal MST‐assisted method to overcome the low‐crystallinity limitation of high‐mobility n‐type F4‐TCNQ. The use of an MST facilitates the lateral packing of F4‐TCNQ molecules through *π*–*π* interactions between the cyclohexadiene of the F4‐TCNQ molecule and the exposed benzothiophene of C8‐BTBT at the step edge. This greatly lowers the nucleation barrier of F4‐TCNQ molecules in the in‐plane stacking direction and efficiently decreases the nucleation density simultaneously. As a result, highly crystalline F4‐TCNQ thin films with large‐sized, compact, and oriented grains were obtained for the first time, leading to remarkably high electron mobilities up to 2.58 cm^2^ V^−1^ s^−1^, which are 10^7^ times greater than those of F4‐TCNQ thin films without a MST. In addition, complementary inverters based on the obtained F4‐TCNQ thin films and the MST exhibit voltage gains as high as 112.6 V V^−1^ and SNMs greater than 89.3% with very good uniformity. Our findings demonstrate that a MST, which enables a substantial increase in the crystallinity of organic thin films, can be utilized to improve the performance of organic electronic devices and to expand their applications.

## 4 Experimental Section

4.1

4.1.1

##### Materials and Solution Preparation

C8‐BTBT and F4‐TCNQ powders were purchased from Luminescence Technology Corp. An amorphous insulating polymer, polystyrene (PS) (Mw = 2000 kDa), was purchased from Sigma–Aldrich Co., Ltd. A mixed semiconductor solution was prepared by dissolving 5 mg of C8‐BTBT and 10 mg of PS in 1 mL of toluene (high performance liquid chromatography grade, ≥99.8%, Adamas‐beta). Divinyltetramethyldisiloxane BCB (CYCLOTENE 3022‐35) was purchased from Dow Chemicals.

##### Fabrication of C8‐BTBT MSTs

In this study, a 300 nm thick thermally grown SiO_2_/Si wafer was used as the substrate. The substrate was spin‐coated with BCB at 500 rpm for 8 s, followed by centrifugation at 3500 rpm for 30 s. The coated substrate was then prebaked at 160 °C for 30 min and annealed at 260 °C for 2 h. The gap between the blade and substrate surface was ≈0.2 mm, and the angle between them was 30°. Subsequently, 2 μL of the C8‐BTBT:PS mixture was added to the gap between the blade and the substrate, and the mixture was then sheared at 300 μm s^−1^ at room temperature.

##### Fabrication and Measurements of Devices

A total of 6 nm of F4‐TCNQ was evaporated thermally at 0.3 Å s^−1^ under a vacuum chamber pressure of 1 × 10^−9^ bar. Then, 50 nm silver (Ag) was thermally evaporated at 0.5 Å s^−1^ under a vacuum chamber pressure of 2 × 10^−9^ bar through a shadow mask with channels L and W of 50 and 500 μm, respectively, to define the S/D electrodes. The gate capacitance per unit area (*C*_*i*_) of the C8‐BTBT/BCB/SiO_2_ dielectric was measured to be 4.70 nF cm^−2^ (Figure S16, Supporting Information). All the devices were measured by using a semiconductor parameter analyzer (Keithley SCS‐4200) with a probe station in air at room temperature. For the measurement of device stability, the devices were kept in a moistureproof cabinet with a humidity of ≈10% at 20 °C.

##### Characterization

The morphologies of the C8‐BTBT MSTs were characterized using a cross‐polarized optical microscopy (Olympus BX51) and AFM (tapping mode, Cipher S). The surface morphology of the F4‐TCNQ thin film on BCB‐coated SiO_2_ and the crystalline F4‐TCNQ thin film on C8‐BTBT MST were characterized using AFM (tapping mode, Cipher S). The crystal orientation of the C8‐BTBT MSTs was confirmed using in‐plane grazing incidence X‐ray diffraction (GIXRD) (Bruker D8 DISCOVER X‐ray diffractometer). The crystallinity of the C8‐BTBT crystals and F4‐TCNQ thin film with C8‐BTBT MST was confirmed using out‐of‐plane GIXRD (Bruker D8 DISCOVER X‐ray diffractometer). UV–vis absorption spectra of C8‐BTBT MST and the F4‐TCNQ thin film with C8‐BTBT MST were obtained using a UV–vis–ultraviolet‐visible‐near infrared  spectrophotometer (Perkin–Elmer Lambda 950).

##### Details of DFT Calculations

First‐principles were employed to perform spin‐polarization DFT calculations within the generalized gradient approximation using the Perdew–Burke–Ernzerhof formulation. Projected augmented wave potentials were chosen to describe the ionic cores, and valence electrons were taken into account using a plane wave basis set with a kinetic energy cutoff of 520 eV. The systems were completely relaxed until the energy and force converged to 10^−6^ eV and 0.02 eV Å^−1^, respectively. In the calculations, a *k*‐point mesh with a spacing of ≈ 0.03 Å^−1^ was adopted. The long‐range interactions were considered a correction in all the calculations. The tilt angle was defined by measuring the angle between the cyclohexadiene plane of F4‐TCNQ and the benzothiophene plane of C8‐BTBT. The binding energy *E*_b_ was calculated from the formula: *E*_b_ = *E*_(C8‐BTBT)_ + *E*_(F4‐TCNQ)_ − *E*_(F4‐TCNQ@C8‐BTBT)_, where *E*_(C8‐BTBT)_, *E*_(F4‐TCNQ)_, and *E*_(F4‐TCNQ@C8‐BTBT)_ are total energies of isolated C8‐BTBT, isolated F4‐TCNQ, and F4‐TCNQ with C8‐BTBT, respectively. The total energy was obtained from self‐consistent calculations.

## Conflict of Interest

The authors declare no conflict of interest.

## Supporting information

Supplementary Material

## Data Availability

The data that support the findings of this study are available from the corresponding author upon reasonable request.
