# Supplementary material for: Large‐Area Deposition of Highly Crystalline F4‐Tetracyanoquinodimethane Thin Films by Molecular Step Templates
Source: Small Sci. 2024 Apr 12;4(7):2400038. doi: 10.1002/smsc.202400038 (PMC11935238; doi:10.1002/smsc.202400038)
Supplement: Supplementary file 1 — Supplementary Material [file SMSC-4-2400038-s001.pdf]

Supporting Information

Large-Area Deposition of Highly Crystalline F4-Tetracyanoquinodimethane Thin Films by Molecular Step Templates

Fengquan Qiu, Wei Deng, Xinmin Shi, Dewen Ai, Xiaobin Ren, Anyi Dong, Xiujuan Zhang, and Jiansheng Jie**

F. Q. Qiu, Prof. W. Deng, D. W. Ai, X. B. Ren, A. Y. Dong, Prof. X. J. Zhang, and Prof. J. S. Jie
Institute of Functional Nano & Soft Materials (FUNSOM)
Jiangsu Key Laboratory for Carbon-Based Functional Materials & Devices
Soochow University
Suzhou, Jiangsu 215123, P. R. China
E-mail: dengwei@suda.edu.cn; jsjie@suda.edu.cn

X. M. Shi, Prof. J. S. Jie
Macao Institute of Materials Science and Engineering (MIMSE)
MUST-SUDA Joint Research Center for Advanced Functional Materials
Macau University of Science and Technology
Taipa, Macau SAR 999078, P. R. China

Keywords: fluorinated tetracyanoquinodimethane; crystalline organic thin film; n-channel organic semiconductor; organic thin-film transistors; organic complementary inverters

Table S1. Surface energies of C8-BTBT and F4-TCNQ determined by Owens–Wendt–Kaelble and Young's equations (γ is the surface energy. The superscripts "*d*" and "*p*" refer to the dispersive and the polar components, respectively).

Materials	Contact angle (°)		Surface energy (mN m^{-1})		
	Deionized water (DI)	Ethylene glycol (EG)	γ^d	γ^p	γ
C8-BTBT	109	83.6	25.97	0.01	25.98
F4-TCNQ	97.7	73.7	23.6	1.48	25.08

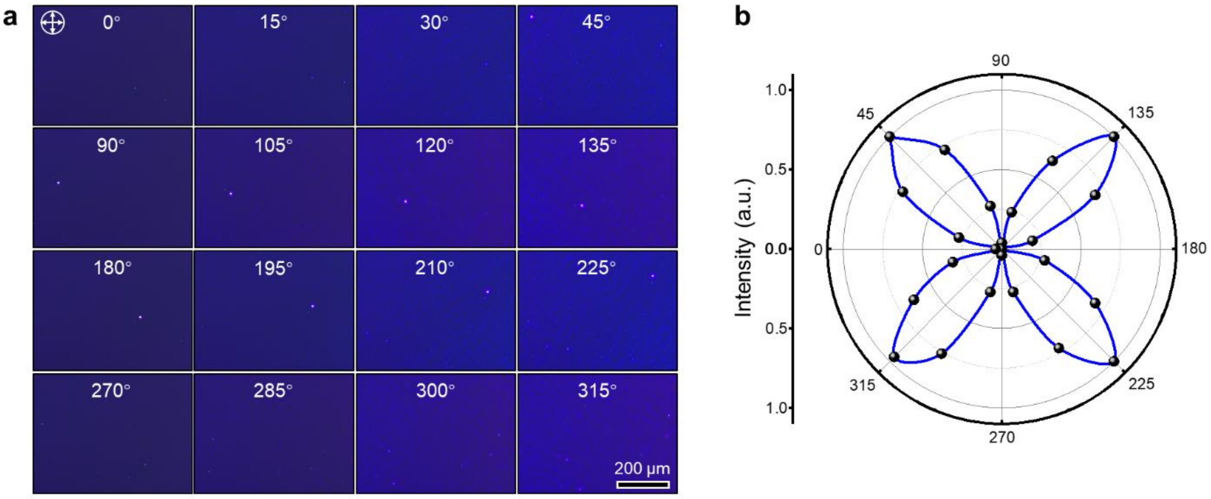

Figure S1. (a) Cross-polarized optical microscope (CPOM) images of the C8-BTBT MST at various polarization angles. (b) Normalized intensity of the C8-BTBT MST under one cycle of polarization angle rotation.

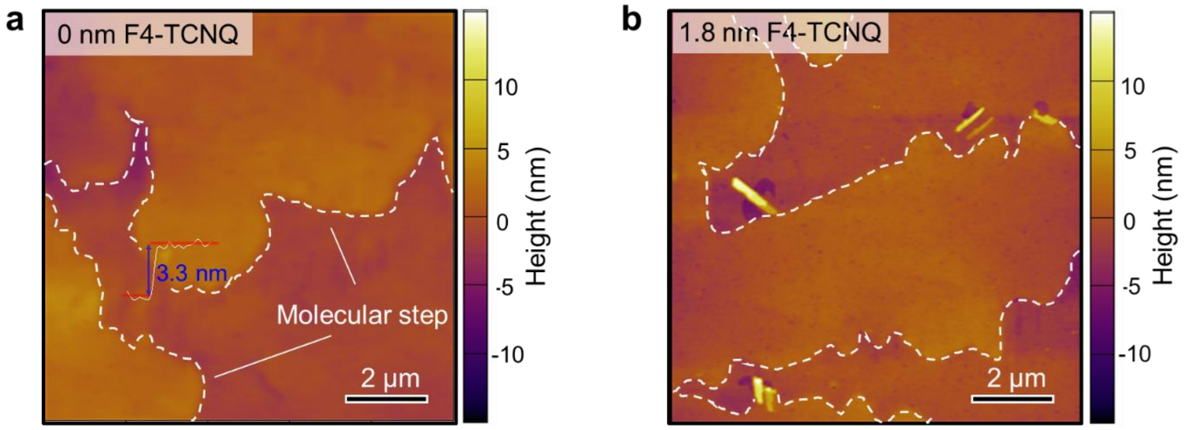

Figure S2. (a, b) AFM topography of C8-BTBT and 1.8 nm F4-TCNQ on the MST with an area of 10 × 10 μm.

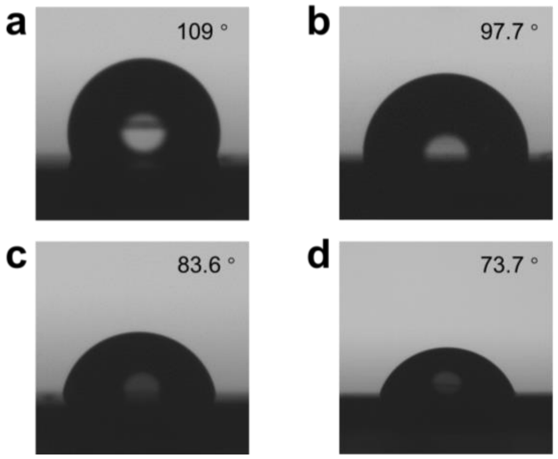

Figure S3. Contact angle measurements of (a, c) C8-BTBT and (b, d) F4-TCNQ surfaces using both deionized water and ethylene glycol.

To determine the surface energies of C8-BTBT and F4-TCNQ, we measured the contact angles on the C8-BTBT and F4-TCNQ surfaces using deionized (DI) water and ethylene glycol (EG) as the probe liquids, whose surface energies are already known. We then calculated the dispersion and polar components of the surface energies for their surfaces using equation（1）:

$$(1 + cos\,\theta)\gamma_l = 4\left[\gamma_l^d\gamma_s^d/\left(\gamma_l^d + \gamma_s^d\right) + \gamma_l^p\gamma_s^p/\left(\gamma_l^p + \gamma_s^p\right)\right] \quad 1$$

which is derived from the Owens-Wendt-Kaelble and Young's equations. Where θ is the equilibrium contact angle made by each liquid on the solid surface, γ is the surface energy. The superscripts d and p refer to the dispersive and the polar components, respectively, and the subscripts l and s refer to the liquid and solid, respectively. The results of C8-BTBT and F4-TCNQ surface are 25.98 mN m^{-1} and 25.08 mN m^{-1}.

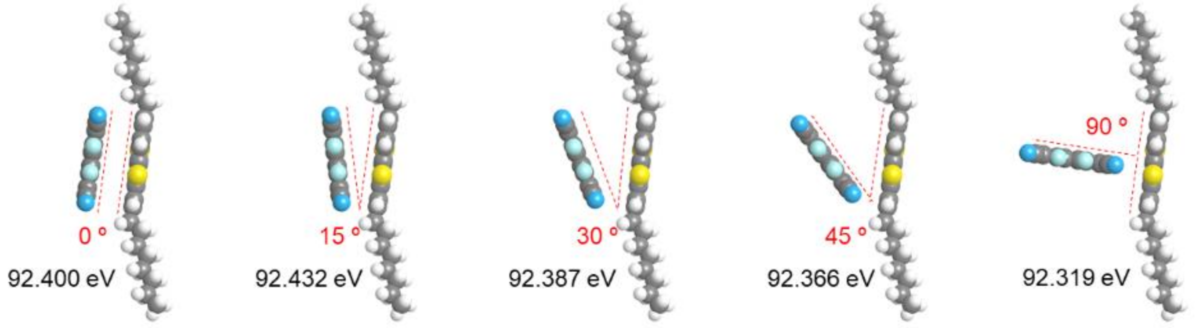

Figure S4. DFT calculations of the structure of F4-TCNQ molecule on C8-BTBT molecular step.

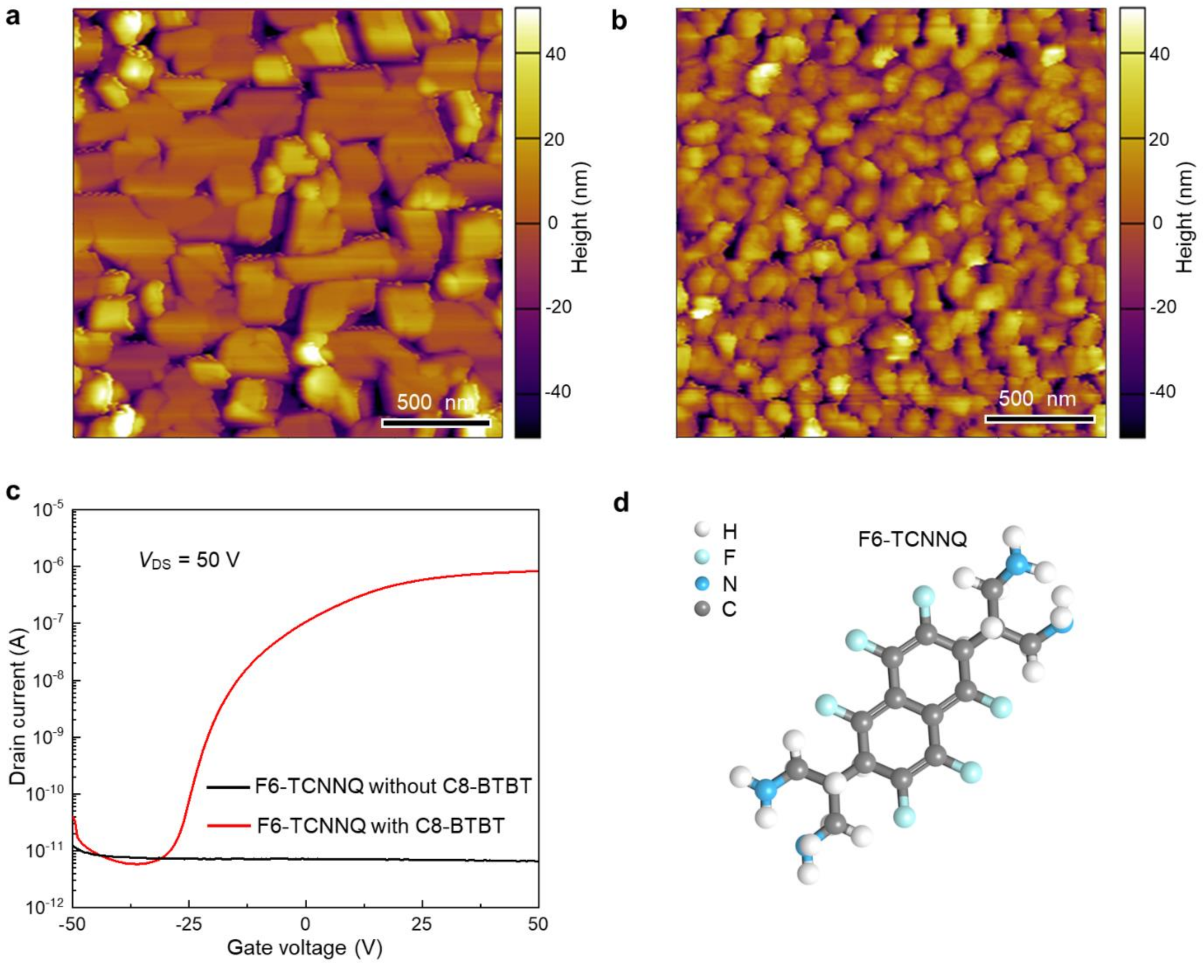

Figure S5. (a, b) AFM topography of the resulting F6-TCNNQ thin films with and without MST. (c) Transfer characteristics of the F6-TCNNQ thin film based OTFTs with C8-BTBT and without C8-BTBT. (d) Ball-and-stick model of the F6-TCNNQ molecule.

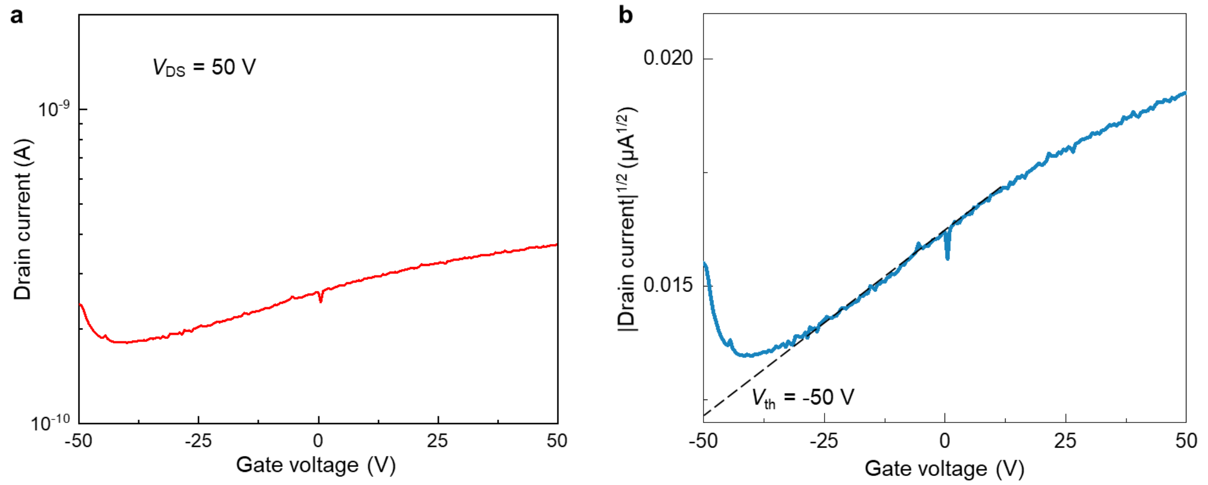

Figure S6. (a) Transfer characteristics of the F4-TCNQ crystalline film without C8-BTBT MST based OTFT. (b) V_{th} of the device.

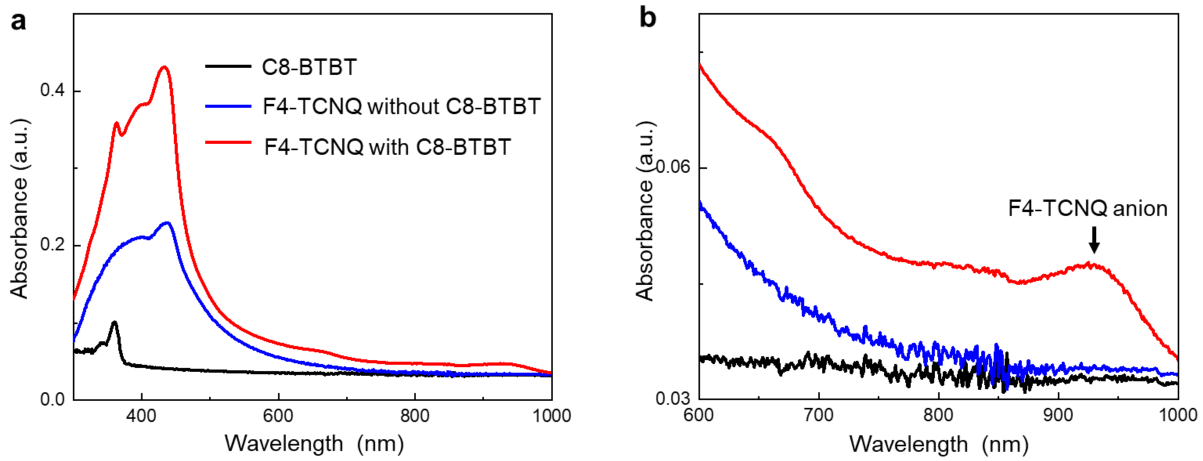

Figure S7. (a,b) UV–Vis absorption spectroscopy of pure C8-BTBT, pure F4-TCNQ, and F4-TCNQ grown on the C8-BTBT MST.

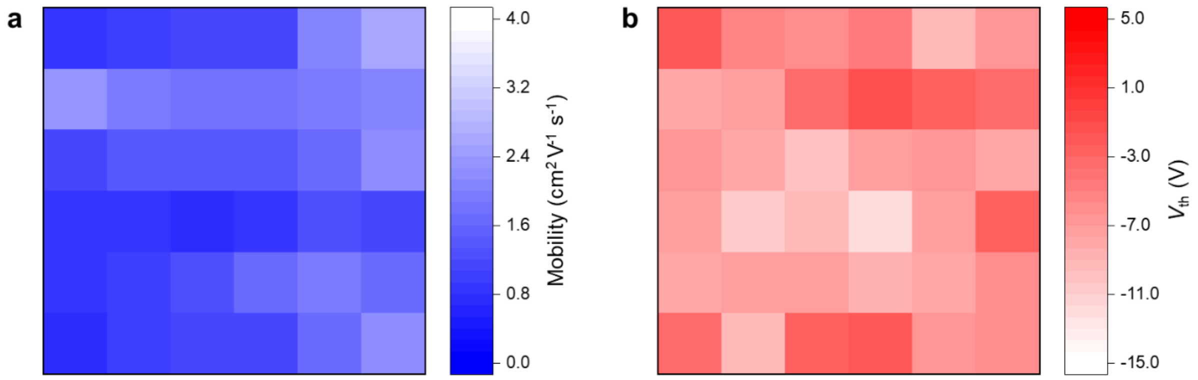

Figure S8. Spatial distribution of electrical characteristics the 36 OTFT array fabricated from the F4-TCNQ crystalline thin films.

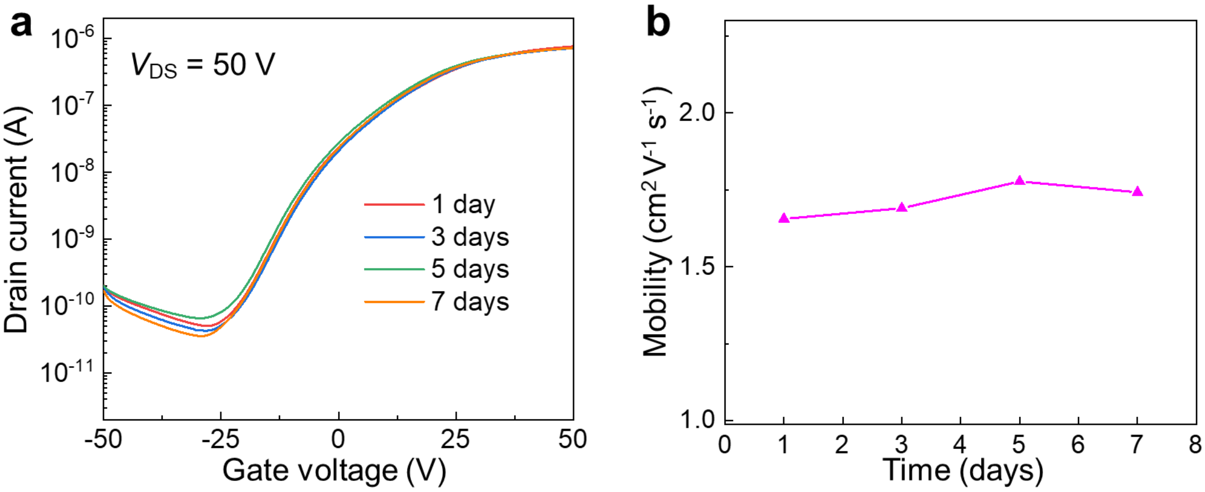

Figure S9. (a) Transfer characteristics of a F4-TCNQ OTFT with MST after different storage days (humidity 50%-70%). (b) Mobility vs. storage time.

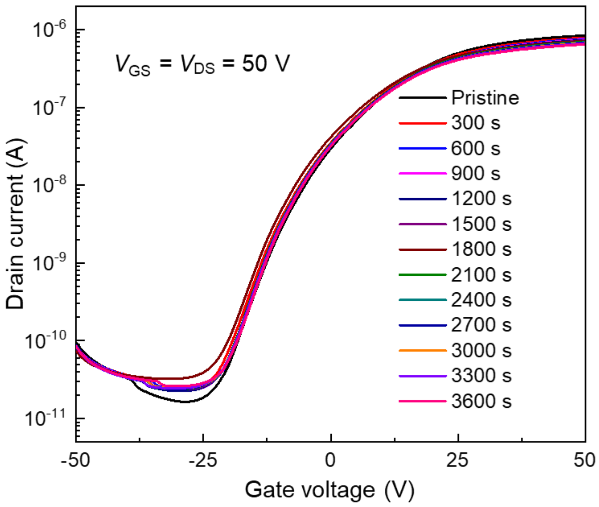

Figure S10. Bias stress stability measurement of the F4-TCNQ OTFT under V_{DS} = V_{GS} = 50 V.

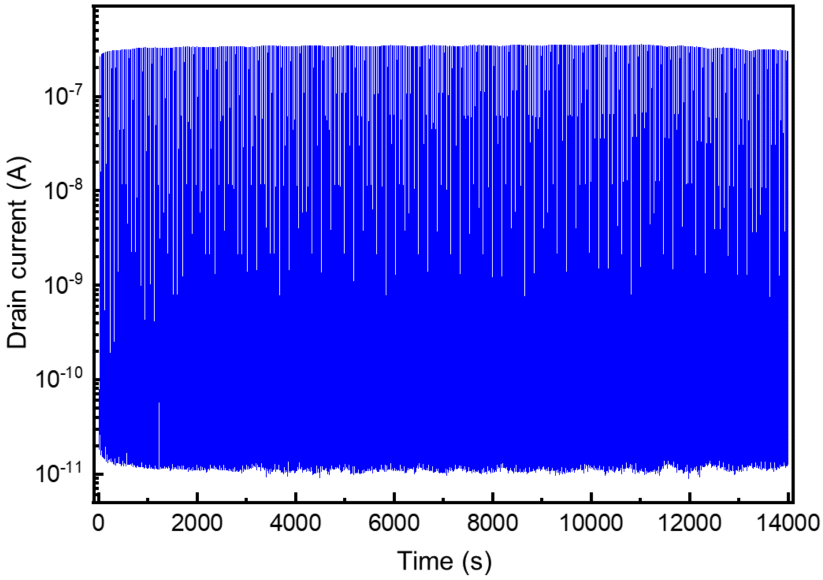

Figure S11. Operating cycle stability of the n-channel OTFT. The device switched continuously on- and off-state condition over a period of 14000 s under V_{DS} = 50 V in air.

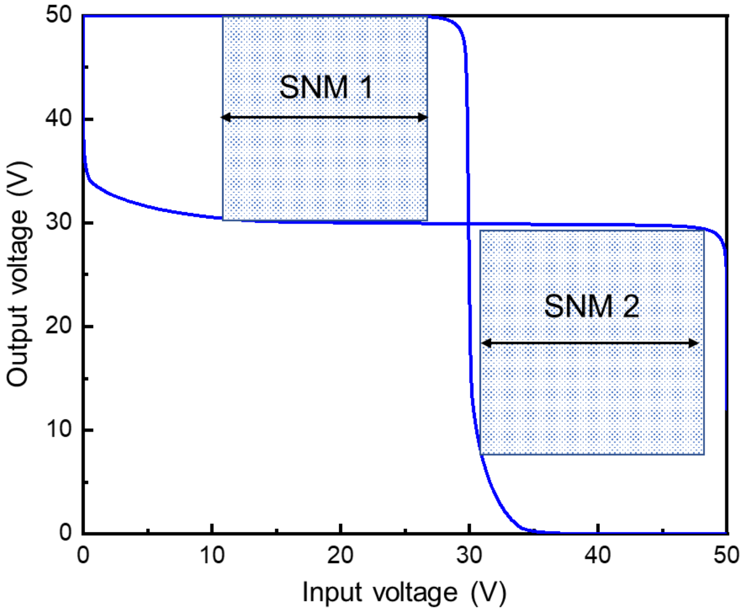

Figure S12. Extraction of SNM. A basic understanding of the SNM is obtained by drawing and mirroring the inverter characteristics and then finding the maximum square between them.

$$SNM = \frac{\text{Length of the square}}{\frac{1}{2}V_{\mathrm{DD}}}$$

Butterfly inverter curves for SNM calculation at V_{DD} = 50 V.

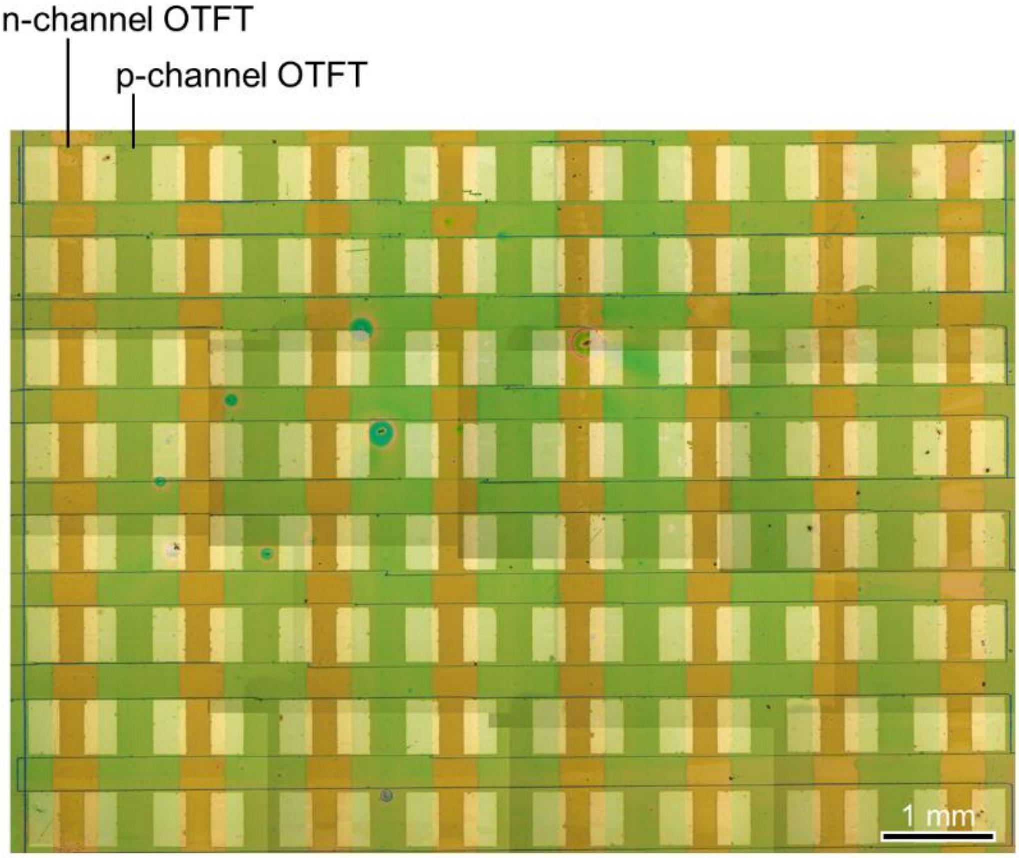

Figure S13. Optical microscope images of complementary inverters over a centimetre scale.

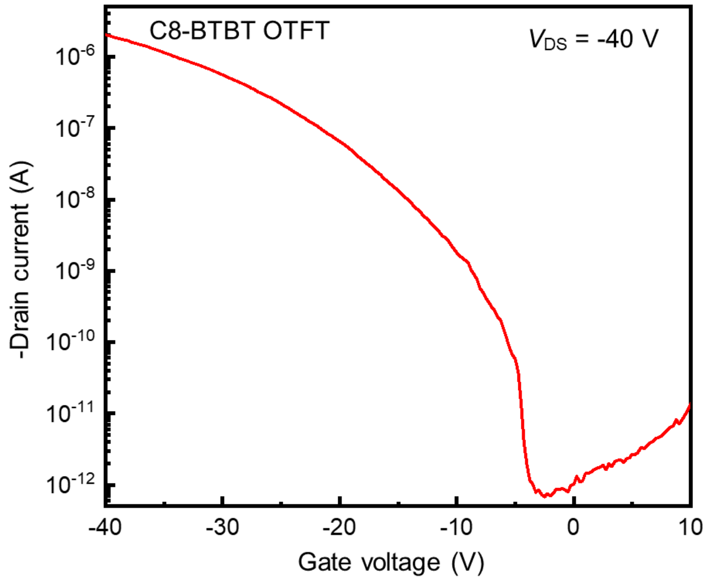

Figure S14. Transfer characteristics of the C8-BTBT MST based OTFT.

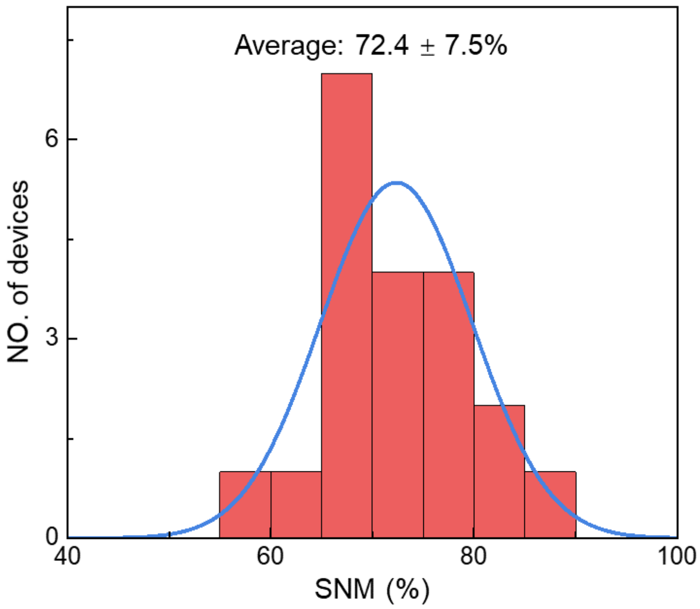

Figure S15. Statistical histograms of SNM. SNM extracted from 20 inverters.

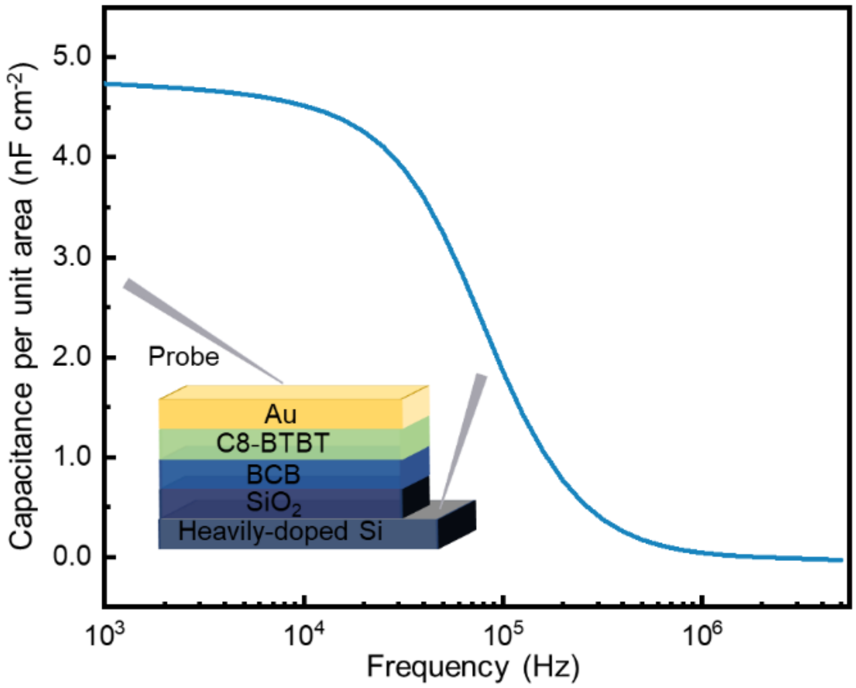

Figure S16. Capacitance per unit area (C_i) of the C8-BTBT/BCB/SiO_2 gate dielectric as a function of frequency.
